# Magnetoencephalography-based approaches to epilepsy classification

**DOI:** 10.3389/fnins.2023.1183391

**Published:** 2023-07-12

**Authors:** Ruoyao Pan, Chunlan Yang, Zhimei Li, Jiechuan Ren, Ying Duan

**Affiliations:** ^1^Faculty of Environment and Life, Beijing University of Technology, Beijing, China; ^2^Department of Internal Neurology, Tiantan Hospital, Beijing, China; ^3^Beijing Universal Medical Imaging Diagnostic Center, Beijing, China

**Keywords:** MEG, epilepsy, classification, deep learning, machine learning

## Abstract

Epilepsy is a chronic central nervous system disorder characterized by recurrent seizures. Not only does epilepsy severely affect the daily life of the patient, but the risk of premature death in patients with epilepsy is three times higher than that of the normal population. Magnetoencephalography (MEG) is a non-invasive, high temporal and spatial resolution electrophysiological data that provides a valid basis for epilepsy diagnosis, and used in clinical practice to locate epileptic foci in patients with epilepsy. It has been shown that MEG helps to identify MRI-negative epilepsy, contributes to clinical decision-making in recurrent seizures after previous epilepsy surgery, that interictal MEG can provide additional localization information than scalp EEG, and complete excision of the stimulation area defined by the MEG has prognostic significance for postoperative seizure control. However, due to the complexity of the MEG signal, it is often difficult to identify subtle but critical changes in MEG through visual inspection, opening up an important area of research for biomedical engineers to investigate and implement intelligent algorithms for epilepsy recognition. At the same time, the use of manual markers requires significant time and labor costs, necessitating the development and use of computer-aided diagnosis (CAD) systems that use classifiers to automatically identify abnormal activity. In this review, we discuss in detail the results of applying various different feature extraction methods on MEG signals with different classifiers for epilepsy detection, subtype determination, and laterality classification. Finally, we also briefly look at the prospects of using MEG for epilepsy-assisted localization (spike detection, high-frequency oscillation detection) due to the unique advantages of MEG for functional area localization in epilepsy, and discuss the limitation of current research status and suggestions for future research. Overall, it is hoped that our review will facilitate the reader to quickly gain a general understanding of the problem of MEG-based epilepsy classification and provide ideas and directions for subsequent research.

## 1. Introduction

Epilepsy is a chronic central nervous system disorder with a prevalence of 1–2% of the world’s population (Mormann et al., 2007). Patients with epilepsy have a three times higher risk of premature death compared to the normal population, however, if timely and correct diagnoses and treatments are obtained in the early stage of the disease, 70% of patients may be effectively controlled (Beghi et al., 2019). Therefore, it is particularly important to reduce the rate of misdiagnosis and underdiagnosis of epilepsy.

Epilepsy is usually caused by abnormal neuronal discharges and is characterized by recurrent seizures. Suddenly recurrent and transient perceptual or behavioral disturbances due to hyper synchronization of cortical neuronal networks, where individuals experience prolonged abnormal discharges in the brain. Seizures are classified according to their clinical presentation as partial or focal, generalized, or unilateral (James, 1997; Tzallas et al., 2007, 2009). Focal seizures involve only a part of the cerebral hemisphere and produce symptoms in the corresponding part of the body or in some associated mental function. Generalized seizures involve the entire brain, producing bilateral motor symptoms and usually loss of consciousness.

One of the diagnostic tools for epilepsy is Magnetoencephalography (MEG) (Hamandi et al., 2016), which is a neurophysiological examination procedure that uses a superconducting quantum interference device (SQUID) to measure brain signals for the diagnosis of many neurological disorders such as Parkinson’s disease (Olde Dubbelink et al., 2014), Alzheimer’s disease and autism (Stam, 2010; O’Reilly et al., 2017). MEG is a non-invasive whole-head neuroimaging modality that uses a highly sensitive magnetometer and gradiometer to record magnetic fields associated with postsynaptic neuronal currents within brain cells (Cohen and Cuffin, 1983). People generally use EEG to diagnose whether a patient has epilepsy clinically. And the EEG is widely used, the examination is affordable, and there is extensive and in-depth research accumulation in the field of research EEG. However, EEG with high temporal resolution and insufficient spatial resolution can suffer from the problem of inaccurate source localization. For reviews on EEG source localization/source estimation source localization/estimation can be found in the literature (Wennberg et al., 2011; Wennberg and Cheyne, 2014). On the other hand, the magnetic signal measured by MEG can pass through the dura, skull, and scalp relatively undistorted and has a higher signal-to-noise ratio (SNR_MEG_ = 2.2 db, SNR_EEG_ < 1 db) and spatial resolution (SR_MEG_ = 2–3 mm, SR_EEG_ = 7–10 mm) than EEG (Fred et al., 2022). Thus, MEG can exactly compensate for the lack of EEG and play a better role in functional localization. Complete excision of the irritative zone defined by the MEG has prognostic significance for postoperative seizure control (Fischer et al., 2005). Magnetic resonance imaging (MRI) re-evaluation guided by MEG helps to detect previously unidentified lesions (Moore et al., 2002), and interictal MEG is superior to scalp EEG and can provide additional, decisive localization information (Papanicolaou et al., 2005). Intracranial electroencephalography (iEEG), stereo-electroencephalography (SEEG), and electrocorticography (ECoG) are invasive tests that are considered the gold standard in the diagnosis of epilepsy. Among them, iEEG is an electrode surgically implanted in the patient’s brain to obtain a continuous recording of local field potentials for several hours (Lachaux et al., 2003), and this continuous data provides valuable information about seizures and anatomical seizure areas (Balaji and Parhi, 2022), as well as local brain tissue stimulation applied through these electrodes. iEEG can be broadly divided into SEEG, which places electrodes in deep brain tissue, and ECoG, which places electrodes in the cerebral cortex. The review of the use of ECoG to assess the location of epileptic pathological activity can be found in the reference (Crone et al., 2006), and the review of studies on the localization of epileptic lesions using SEEG can be found in the reference (Ramantani et al., 2013; Bartolomei et al., 2017). However, MEG, as a non-invasive method, can play a full role in pre-screening and can avoid in advance the harm that some patients receive during invasive examinations. Therefore, we need to vigorously develop pre-screening and then use invasive instruments such as iEEG for validation in the face of more severe situations. Functional magnetic resonance imaging (fMRI) techniques have been applied as a well-established noninvasive brain imaging method to demonstrate intrinsic abnormalities in various types of epilepsy (Vlooswijk et al., 2010), which can indirectly reflect brain activity and possess high spatial resolution, however, fMRI has a temporal low resolution is relatively. In contrast, MEG source localization has been shown to improve the possibility of sampling the seizure onset zone (SOZ) in iEEG assessment, helping to determine the location of iEEG electrode placement (Stefan et al., 2011): non-invasive localization of the sources of MEG epileptiform discharges are not necessarily detected by simultaneous EEG, whereas MEG combined with detailed MRI and functional imaging studies can be effectively used for epilepsy surgery planning (Rosenow and Lüders, 2001; Seo et al., 2011). MEG has been recognized as an effective tool for diagnosing epilepsy and finding the location of cortical pathological activity or damage in epileptic foci (Burns et al., 2014). In patients with epilepsy, MEG is used in two main phases of treatment (Englot et al., 2015; Stefan and Trinka, 2017): (i) to localize areas of the brain that generate abnormal electrical activity causing neurological disorders, and (ii) to assess the effectiveness of surgery.

The most common methods of detecting epilepsy from brain signals are through visual inspection and manual annotation. MEG is a laborious and time-consuming task for physicians due to the number of sensors (Halford, 2009; Chahid et al., 2020), the complex pre-processing required to extract cortical signals, and the experience required to classify the various waveforms. Due to the limited number of MEG instruments available worldwide and their high cost, the use of MEG signals for epilepsy diagnosis and abnormality detection is still rare, although in recent years there has been a growing number of scholars who have analyzed MEG signals for activity (van Diessen et al., 2015). Therefore, the use of computer-aided detection is of great importance in solving the problem of clinical diagnosis of neurological disorders.

## 2. Materials and methods

In this section, we describe the use of classification in relation to epileptic disorders. We searched Web of science, Google scholar and other websites for over a hundred papers by searching for the keywords including MEG (or Magnetoencephalography), epilepsy, classification. Then, more than ten studies related to the tpoic were selected (i.e.,MEG data using classification for epilepsy diagnosis), which are described in the Section “2.1. The methods for epileptic patients classification based on MEG signals”. In addition, we added the keyword of spike/HFO to the previous search in order to illustrate the use of classification using MEG signals for the auxiliary localisation of epilepsy, and after removing any irrelevant content on the topic there were more than ten research-based papers, which are described in the Section “2.2. Epilepsy-assisted localization methods using MEG signals”. To highlight the topic of this review, we will focus on studies related to the classification/diagnosis of epileptic disorders on MEG modalities, and we will outline the studies on the use of MEG signals for auxiliary localisation of epilepsy.

**FIGURE 1:**
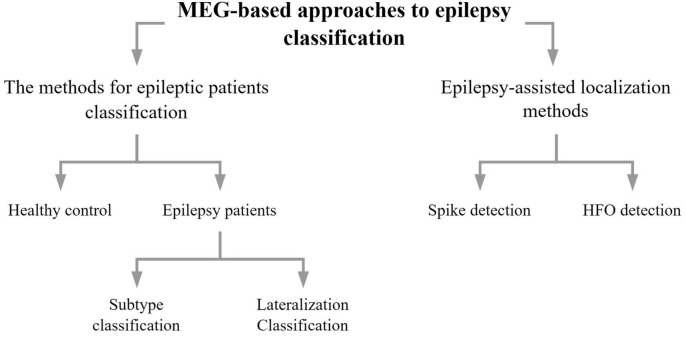
Diagram of methods concerning epilepsy classification based on MEG data.

As can be seen in Figure 1, the methods section of this review describes methods concerning epilepsy classification using data from MEG, addressing mainly epilepsy diagnosis (Section “2.1. The methods for epileptic patients classification based on MEG signals”) and epilepsy auxiliary localization (Section “2.2. Epilepsy-assisted localization methods using MEG signals”). Furthermore, epilepsy diagnosis is divided into healthy control v.s. epilepsy patients, and epilepsy patients can be divided into epilepsy subtypes or laterality. The auxiliary localization of epilepsy can be divided into methods of spike detection and HFO detection. In this paper we discussed them in the order of machine learning, deep learning, and other classification methods.

### 2.1. The methods for epileptic patients classification based on MEG signals

Direct signal-based computer-aided diagnosis (CAD) of epilepsy is a relatively intuitive method. The vast majority of these studies have been carried out using machine learning classification methods, which broadly consist of three steps: signal pre-processing, feature extraction and classification, while only a few studies have performed classification by deep learning. In this section, we review the classification methods that have been used so far for epilepsy diagnosis using deep learning for MEG signals. An overview of all the studies mentioned in this section can be viewed in Tables 1, 2.

**TABLE 1:** The methods for classifying epilepsy using machine learning in MEG data.

References	Moda-lity	Problem that was solved in that study	Database					Data acquisition				Source locali-zation	Fea-tures	Classifi-cation	Performance metrics		
			**Sample size**	**Age range**	**Sex** **(M:F)**	**Source**	**Total dura-tion**	**Cha-nnels**	**Seg-ment/** **epoch** **length**	**Fre-quency samp-ling**	**Pre-process-ing**				**Sensiti-vity** **(%)**	**Specifi-city** **(%)**	**Accu-racy** **(%)**
Khalid et al., 2015	MEG	EP vs HC	18 EPs, 15 HCs	/	/	KFMC	15 min	more than 300	/	1,000 Hz	/	/	Standard deviation	LDA	94.4	100	95.7
Khalid et al., 2016a	MEG	EP vs HC	35Eps, 35HCs	/	/	KFMC	15 min	306	/	1,000 Hz	/	/	Energy of the Delta and Theta component	Threshold method	96.66	98.66	/
Matsubara et al., 2018	MEG	mTLE vs HCs	25 left mTLE, 14 right mTLE, 32 HCs	20–68	females	Kyushu University	at least 120 evoked responses were counted	306	/	1,000 Hz	band-pass filter: 0.1–330 Hz	minimum norm estimate (MNE) software	phase-locking factor (PLF) and phase-locking value (PLV)	LDA	82.1	81.3	81.7
		left TLE vs right TLE													92	92.9	92.3
Wu et al., 2018	MEG	left / right TLE vs HCs	15 left/15 right TLEs, 15 HCs	15–62	/	Nanjing Brain Hospital, Nanjing Medical University	30 min	275	120 s	1,200 Hz	band-pass filter: 1–4 Hz	standardized low resolution brain electro-magnetic tomography (sLORETA) was based on minimum-norm estimation (MNE)	nodal degree, betweenness centrality, and nodal efficiency	RBF-SVM	/	/	77.38
		left TLE vs right TLE													/	/	88.1
Alotaiby et al., 2019	MEG	EPs vs HCs	32 EPs, 32 HCs	/	/	KFMC	≈ 19 min	306	1 min	1,000 Hz	band-pass filter: 0.03–330 Hz	/	8 statistical features	RBF-SVM	99.35	95.47	/
Gautham et al., 2022	MEG	left TLE vs HCs	54 TLE, 21 HCs	15–37	36:18	the MEG Research Centre at NIMHANS, Bangalore, India	5 min	306	/	2,000 Hz	down-sampled to 500 Hz	beamformer	phase amplitude coupling (PAC)	SVM	/	/	92.92
		right TLE vs HCs													/	/	93.54
		left TLE vs right TLE													/	/	92.04
Wang et al., 2022	MEG	CPS vs SPS	16 SPS, 16 CPS	17–38	13:19	Nanjing Brain Hospital, Nanjing Medical University	40 min	275	120 s	1,200 Hz	low-pass filter:70 Hz, high-pass filter: 1,000 Hz, notch-filter: 50 Hz, down-sampled to 100 Hz	partial canonical correlation/ coherence (PCC), Fieldtrip	resting state functional connectivity features	SVM	81.1	81.54	81.37
Soriano et al., 2017	MEG	EPs vs HCs	14 frontal focal EPs, 14 idiopathic generalized EPs, 14 HCs	16–52	1:1	the University General Hospital of Ciudad Real	10 min	306	5 s	1,000 Hz	band-pass filter: 0.1–330 Hz	/	total and relative power	ELM	93	86	90
		generalized vs focal epilepsy											spectral densities (PSD), the phase-locking value (PLV) and the phase-lag index (PLI)		1	86	93
Bhanot et al., 2022	MEG	localize the brain region from where the seizure originated	15 EPs	14–34	9:6	the MEG Research Centre at NIMHANS, Bangalore, India	12 min	306	/	2,000 Hz or 50,000 Hz	band-pass filter: 0.1–100 Hz, down-sampled to 250 Hz	/	short-time permutation entropy (STPE), gradient of STPE (GSTPE), short-time energy (STE), and short-time mean (STM)	RUSBoost	93	93	93.4

EPs, Epileptic patients; HCs, Healthy controls; TLE, temporal lobe epilepsy; SPS, simple partial seizure; CPS, complex partial seizure; SCI, spinal cord injury patients; NNI, National Neuroscience Institute; KFMC, King Fahad Medical City, Riyadh, Saudi Arabia.

**TABLE 2:** The methods for classifying epilepsy using deep learning in MEG data.

References	Moda-lity	Problem that was solved in that study	Database					Data acquisition				Source locali-zation	Fea-tures	Classi-fication	Performance metrics		
			**Sample size**	**Age range**	**Sex** **(M:F)**	**Source**	**Total dura-tion**	**Cha-nnels**	**Seg-ment/** **epoch length**	**Fre-quency samp-ling**	**Pre-process-ing**				**Sensiti-vity** **(%)**	**Specifi-city** **(%)**	**Accu-racy** **(%)**
Aoe et al., 2019	MEG	EPs vs HCs	140 EPs, 26 SCIs, 67 HCs	21–86	123:110	Osaka University hospital	4 or 5 min	160	800 ms	1,000 Hz or 2,000 Hz	low-pass filter: 50 Hz, high-pass filter: 1,000 Hz, down-sampled to 1,000 Hz	/	/	MNet	/	/	63.4 ± 12.7
		EPs vs SCIs													/	/	79.8 ± 11.7
		EPs vs SCIs vs HCs													/	/	70.7 ± 10.6
Gu et al., 2020	MEG	SPS vs CPs	32 EPs	20–32	1:1	Nanjing Brain Hospital, Nanjing Medical University	40 min	/	2 min	1,200 Hz	band-pass filter: 0.03–300 Hz	/	/	MSAM	90.8	90.7	83.6
Fujita et al., 2022	MEG	EPs vs HCs	90 Eps, 90 HCs	7–86	93:87	Osaka University hospital	4 or 5 min	160	2,400 ms	1000 Hz or 2,000 Hz	low-pass filter: 500 Hz, high-pass filter: 0.1 Hz, bandstop filter: 60 Hz, down-sampled to 1,000 Hz	/	relative power, functioncal connectivity (FC), phase-amplitude coupling (PAC)	MNet (Aoe et al., 2019)	90	90	90

#### 2.1.1. Machine learning

##### 2.1.1.1. Linear discriminant analysis and threshold method

Linear Discriminant Analysis (LDA) is a classical linear learning method, introduced by Fisher in 1936 for binary classification problems, hence the name Fisher Discriminant Analysis, and is a supervised learning technique for dimensionality reduction. The idea is that given a set of training samples, the samples are projected into a straight line so that the projections of the same samples are as close as possible and the projection points of dissimilar samples are as far apart as possible, i.e. the projection minimizes the intra-class variance and maximizes the inter-class variance.

In 2015, Kahlid et al. first classified epilepsy patients versus healthy subjects using the LDA approach (Khalid et al., 2015). They found that the subjects’ MEG data followed a normal distribution within eight brain regions (right frontal, left frontal, right temporal, left temporal, right parietal, left parietal, right occipital, and left occipital), but with different standard deviations. Therefore, the standard deviation of the fitted normal distribution for these eight brain regions was used as the feature vector and then LDA classification was performed, resulting in a sensitivity of 94.4% and a specificity of 100%. This study also provided an initial exploration for subsequent epilepsy detection based on the MEG signal.

Another new attempt was made by Khalid et al. (2016a), which they observed that the ratio of Delta-band and Theta-band energy was influenced by epilepsy versus healthy subjects. Therefore, by calculating the band energies of the Delta and Theta band spectrum, and then calculating their ratio (R = D/T), and then comparing it to a pre-determined threshold (Tr), which is the average of the minimum value of the energy ratio in epileptic patients and the maximum value of the energy ratio in healthy subjects, to determine whether the MEG data considered was epileptic, yielded mean sensitivity of 96.66% and specificity of 98.66%.

MEG has rarely been applied to solve the problem of classifying temporal lobe epilepsy (TLE). Matsubara et al. made an attempt in 2018 (Matsubara et al., 2018), and found that neural synchronization induced by unilateral pure tone stimulation provided useful information for determining the lateralization of mesial temporal lobe epilepsy (mTLE). This study applied monaural auditory stimuli to subjects and then analyzed auditory evoked magnetic fields (AEFs) data using source estimates of M100 responses in bilateral auditory cortices (ACs), and used the phase-locking factor (PLF) and phase-locking value (PLV) to assess the neural synchronization of the auditory cortex. While, the PLF represents an index of the inter-trial synchronous change from a local brain region, while the PLV indicates an index of the difference in phase synchronous change between source activities from two different brain regions. Finally, PLFs and PLVs from all neural synchronization data were used for epilepsy diagnosis and laterality diagnosis using LDA. The results showed that the classifier achieved accuracy, sensitivity, and specificity of 81–82%; and for mTLE laterality, 92–93% results were achieved, which is comparable to the results of a study with an EEG (Verhoeven et al., 2018). The results suggest that PLFs are sufficiently sensitive to distinguish between the mTLE group and the healthy controls group (HCs); PLVs show altered functional connectivity between bilateral ACs in patients with right and left mTLE compared to HCs; monaural stimulation reveals AC asymmetry and provides important lateralization information about the epileptic lesion, especially in patients with right mTLE; and LDA works well in this study.

##### 2.1.1.2. Support vector machine

Support Vector Machine (SVM) is a supervised machine learning algorithm proposed in 1964 and has been rapidly developed since the 1990s. It has become one of the most popular machine learning algorithms (Boser et al., 1992), and has been successfully applied as a classifier in different fields. As a classification tool, the SVM technique is flexible, automated, and fast enough to operate in a clinical setting (Focke et al., 2012). SVM algorithms have been applied to measure brain morphology (Rudie et al., 2015), including cortical thickness, volume, curvature, and identifying medial temporal sclerosis (MTS) in TLE patients. SVM has also been used to determine the laterality of TLE epileptogenic foci and diffusion tensor imaging (DTI) structural connectomes (Kamiya et al., 2016). Another study validated the use of SVM for voxel-based MRI classification, where TLE with MTS could be distinguished from TLE without MTS with an accuracy of over 88% (Focke et al., 2012). SVM was also applied to word decoding and MEG recording in schizophrenia (Ince et al., 2008; Chan et al., 2011). In addition, SVM has been used on fMRI to classify TLE patients from healthy subjects (Hao et al., 2022).

It has been shown that unilateral TLE is not a disease with a single focal region, but a network disease (Pittau et al., 2012; Chiang and Haneef, 2014; de Campos et al., 2016), and in addition graph-theoretic metrics are able to summarize network properties with less computational cost than voxel-based and skeleton-based approaches. Wu et al. investigated the problem of unilateral classification for epilepsy based on network properties (Wu et al., 2018). The brain network was first constructed from MEG data, then the connection matrix was obtained to calculate the network parameters associated with the nodes (node degree, node efficiency, and inter-node sex), incorporated into the feature vectors, dimensionality reduction was performed using PCA, and the best feature vectors were fed into the radial basis function kernel SVM (RBF-SVM) for classification. The results found that nodal degree performed best in distinguishing left/right TLE from healthy subjects with accuracy of 80.76 and 75.00%, respectively; betweenness centrality achieved the least accuracy when used as a feature to distinguish left/right TLE from healthy controls, while it performed best in distinguishing left TLE from right TLE with accuracy of 88.10%, which is consistent with its performance on the DTI (Kamiya et al., 2016). This study illustrates the better effectiveness of nodal degree when classifying epilepsy versus controls using brain network features, and the greater clinical value of betweenness centrality when addressing the lateralization of unilateral TLE.

Alotaiby et al. proposed a statistical feature-based MEG signal classification technique (Alotaiby et al., 2019). The multichannel MEG signal was first segmented into time segments of less than 1 minute, and then eight statistical features (maximum, minimum, mean, standard deviation, median, kurtosis, quartile, and skewness) were extracted from the signal, and the features were fed into an SVM classifier for training, with an average sensitivity and specificity of 99.35 and 95.47% for classification. Using the method on the same dataset (mean sensitivity 100%, mean specificity 90.63%) (Khalid et al., 2015), the sensitivity was found to be almost unchanged, while the specificity was increased by 4.84% (Alotaiby et al., 2019).

Tanoue et al. established specific oscillatory distribution and laterality of oscillatory power by analyzing the frequency of unilateral mTLE, calculated the laterality index (LI) for four brain regions (frontal, temporal, parietal, and occipital) in the frequency band (Tanoue et al., 2021), and performed a comparison between subject groups (left mTLE vs HC, right mTLE vs HC). The predictions were then made using an SVM with a linear kernel function, achieving a correct rate of over 91%, a specificity of over 96%, and a sensitivity of 68–75%.

Network disturbance has been shown to exist in multiple frequency bands in the TLE (Bettus et al., 2010; Cataldi et al., 2013), and this is supported by the overlapping nodes of many resting-state networks, where communication between different network elements may be affected by their residual frequencies. Therefore, the optimal function must be included within a given frequency band as well as effective information transfer with other frequency bands. Gautham et al. used the phase-amplitude coupling (PAC) of the resting state MEG to automatically determine the lateralization of TLE lesions (Gautham et al., 2022). PAC assumes that information transfer occurs between the high-frequency power envelope and the low-frequency phase. The phase modulation or entrainment of higher frequency power. There is evidence of specific PAC in physiological recordings as well as during epilepsy (Tort et al., 2010; Nariai et al., 2011; Szczepanski et al., 2014; Amiri et al., 2016). This study used decision tree (DT), SVM-RBF, and naive Bayes (NB) to classify PACs for source-transformed resting states of subject data after feature selection by chi-square test. The results showed that the highest accuracy was achieved between the control and left TLE in terms of the low gamma-low frequency band using SVM (92.92%), between the control and right TLE in terms of the low gamma-low frequency band using DT and SVM (93.54%), and in distinguishing between the left/right TLE using delta-low gamma coupling, theta-low gamma coupling as features and the best results when using the SVM classifier (both 92.04%). It was found that overall PAC values were higher in TLE patients compared to healthy controls. In sub-band analysis, differences were found between controls, left and right TLE: PAC in TLE resting state recordings showed that TLE patients had altered network dynamics on multiple time scales, even in the absence of seizures and interictal discharges. Among the three algorithms used in this study, SVM-RBF provides a non-linear separation between classes for high-dimensional electrophysiological data with small sample sizes, and this classification method is also more straightforward for clinicians.

Wang et al. used differences between functional networks to classify two subtypes of TLE, Simple partial seizure (SPS) and Complex partial seizure (CPS) (Wang et al., 2022). The MEG brain network functional connectivity matrix was constructed and then SVM was used to identify differences in resting state functional connectivity. The results showed that the SVM classification model achieved an average accuracy of 81.37%, sensitivity of 81.1%, and specificity of 81.54% using the best set of 28 features of functional connectivity calculated from the MEG data. The results showed that compared to SPS, CPS patients showed hyper-connectivity in several major regions, including intraparietal sulcus, transverse parietal sulcus of brissaud, middle frontal gyrus, callosal suleus. By comparing the differences in functional connectivity between the SPS and CPS, the pathological basis for the impairment of consciousness and cognitive abnormalities can be explored.

##### 2.1.1.3. Deep learning

Machine learning methods can certainly assist in diagnosis, however, the appropriate features need to be extracted manually. Deep learning methods, on the other hand, can automatically find features and have been successful in other neuroimaging diagnostics, such as MRI imaging of Alzheimer’s disease and brain tumors (Payan and Montana, 2015; Chang et al., 2018).

Aoe et al improved a deep neural network for MNet based on the previous EnvNet-v2 that can classify a variety of neurological disorders based on resting-state MEG signals (Tokozume and Harada, 2017; Tokozume et al., 2017; Aoe et al., 2019). This study attempts to classify MEG signals from spinal cord injury patients, epilepsy patients, and normal controls. MNet is designed to extract the global features of the 160 original MEG signal channels mainly through the first convolutional layer over the entire channel of 64 ms, with the later layers used to extract the time-frequency components of the global features. In this study, the dataset was expanded by slicing. In addition, some of the band power of the 800 ms MEG signal was used as input to the fully connected layer, as these are known classical features that can inform disease classification (Hill et al., 2006; Chaturvedi et al., 2017). The results showed that the classification accuracy was 70.7 ± 10.6% when distinguishing between healthy subjects, epileptic patients, and patients with spinal cord injury, which greatly exceeded the accuracy of classification using SVM when the relative power of the six frequency bands was used as a feature. The classification accuracy was 88.7 ± 9.3% when distinguishing between epileptic and healthy subjects, and accuracy of 79.8 ± 11.7% when distinguishing between epileptic and spinal cord injured patients, both of which also outperformed the SVM classifier. This study is the first to use the MEG signal to classify different neurological disorders based on a single classifier that is not dependent on gender differences and age, and has some generalization, as well as the expanded dataset approach compensates to some extent for the lack of data and has a high specificity in identifying disorders. This study demonstrates that global features of characterized diseases can be successfully extracted using MNet.

Gu et al. proposed a multi-head self-attention model (MSAM) to classify SPS and CPS (Gu et al., 2020). In this case, the original MEG signal was used as input, and a self-attentive mechanism analyzed the effect of the location of the sampled signals to set different weights for the classification algorithm, separating the pre-ictal and interictal periods, and then a multilayer perceptron model was used to extract frequency- and time-domain information for feature extraction and epileptic subtype classification. Experimental results of this study using the MSAM model showed an accuracy of 83.6%, a recall of 90.9%, an accuracy of 90.7%, and an F1 score of 83.4%, significantly outperforming classifiers such as convolutional neural networks, SVM and random forests (RF). This study demonstrates the effectiveness of MSAM in classifying temporal lobe epilepsy subtypes, but further attempts at other datasets, long-term follow-up experiments, and research into the prevention and early treatment of epilepsy are needed.

Yuya et al. hypothesized that resting-state PAC would be different for epileptic patients and healthy subjects in the interictal period (Fujita et al., 2022). This study used the average of the synchronization index (SI) to assess PAC and then used features such as PAC, relative power, and functional connectivity in the δ (1–3 Hz), θ (4–7 Hz), α (8–13 Hz), β (13–30 Hz), low γ (35–55 Hz), and high γ (65–90 Hz) bands, and by individuals or a combination of extracted features followed by classification using MNet (Aoe et al., 2019), to test whether PAC improves discrimination accuracy. The results found that the mean SI was significantly different between epileptic and healthy subjects and that the difference in SI values for theta/low gamma was highest in the temporal lobe, with the highest classification accuracy of 90% when using a combination of PAC and deep learning. This was a slight improvement in accuracy compared to be used only MNet (Aoe et al., 2019).

### 2.2. Epilepsy-assisted localization methods using MEG signals

As previously mentioned, the advantages of MEG in the assisted localization of epilepsy, and both spikes and high-frequency oscillations (HFO) have been clinically shown to be associated with seizures. Therefore, in this section we will review the classification studies of spike detection and high-frequency oscillation detection using the CAD approach. The outline of the studies mentioned in this section can be viewed in Table 3.

**TABLE 3:** The methods of using artificial intelligence for epilepsy-assisted localization in MEG data.

References	Moda-lity	Problem that was solved in that study	Database					Data acquisition				Source locali-zation	Fea-tures	Classifi-cation	Performance metrics		
			**Sample size**	**Age range**	**Sex** **(M:F)**	**Source**	**Total dura-tion**	**Cha-nnels**	**Seg-ment/** **epoch length**	**Fre-quency samp-ling**	**Pre-process-ing**				**Sensiti-vity** **(%)**	**Specifi-city** **(%)**	**Accu-racy** **(%)**
Khalid et al., 2016b	MEG	spike detection	20 Eps	/	/	KFMC	15 min	306	/	1,000 Hz	band-pass filter: 1–50 Hz	/	CSP features	CSP-LDA	91.03	94.21	/
Alotaiby et al., 2017	MEG	spike detection	30 Eps	14–43	22:8	KFMC	15 min	306	100 ms	1,000 Hz	band-pass filter: 1–50 Hz	/	statistical features	KNN	91.75	92.99	/
Khalid et al., 2017	MEG	spike detection	28 Eps	14–43	/	KFMC	15 min	306	100 ms	1,000 Hz	band-pass filter: 1–50 Hz	/	amplitude threshold-based features	Dynamic Time Warping (DTW)	92.45	95.81	/
Chahid et al., 2019	MEG	spike detection	8 Eps, 8 HCs	/	/	KFMC	15 min	306	sliding window of size 100 sample-points with a step of 2 sample-points	1,000 Hz	band-pass filter: 1–50 Hz	/	Semi-Classical Signal Analysis (SCSA) method-based features	SVM	92.52	89.1	90.88
Chahid et al., 2020	MEG	spike detection	8 Eps, 8 HCs	/	/	KFMC	15 min	306	sliding window of size 100 sample-points with a step of 2 sample-points	1,000 Hz	band-pass filter: 1–50 Hz	/	QuPWM-based features	SVM	87	97	/
Sdoukopoulou et al., 2021	MEG+ EEG	spike detection	1 Eps	20	female	/	8 min	304	400 ms	2,400 Hz	band-pass filter: 1–100 Hz	/	EMEG features (statistical, spectral, functional connectivity metrics)	SVM	95.1	90.2	92.8
Kaur et al., 2022	MEG	spike detection	20 EPs	15–52	/	Magnetoence-phalography Center of Xuanwu Hospital of Capital Medical University	60 min	306	10 s	1,000 Hz	band-pass filter: 0.1–500 Hz	/	Phase locking value (PLV)	SVM	/	/	93.8
Zheng et al., 2019	MEG	spike detection	20 focal Eps	10–49	11:9	the Sanbo Hospital of Capital Medical University, Beijing, China	10 min (90 min)	306	300 ms	1,000 Hz	band-pass: 1–100 Hz	/	/	EMS-Net	91.61–99.53	91.60–99.96	91.82–99.89
Hirano et al., 2022	MEG	spike detection	375 EPs	0–79	1:1	Osaka University hospital	4 or 5 min	160	2,048 ms	1,000 Hz or 2,000 Hz	band-pass filter: 3–35 Hz; downsampled: 1,000 Hz	/	/	SE-ResNet + DeepUNet	79.52	99.71	/
Guo et al., 2018	MEG	HFO detection	20 EPs	6–60	1:1	/	60 min	306	2 s	2,400 Hz	band-pass filter: 1–70 Hz, 80–250 Hz, 250–500 Hz; down-sample factor: 10	/	SSAE model-based features	SMO	88.2	91.6	89.9
Guo et al., 2020	MEG	HFO detection	20 EPs	6–60	1:1	/	60 min	306	1,000 ms	4,000 Hz	band-pass filter: 80–250 Hz, 250–500 Hz	/	/	ARF-AttNN	82.6	92.7	89.3
Liu et al., 2020	MEG	HFO detection	20 EPs	6–60	1:1	/	60 min	306	500 ms	2,400 Hz	band-pass filter: 80–250 Hz, 80–500 Hz	/	/	MEGNet	94	/	94
Tanoue et al., 2021	MEG	HFO detection	16 left mTLE, 19 right mTLE	8–71	2:3	Osaka City University Hospital	5 min	160	10 s	1,000 Hz	band-pass filter: 0.3–200 Hz	COH algorithms imple-mented in SPM-12, which is similar to sLORETA	the laterality index (LI) in	SVM	68–75	96	91
Guo et al., 2022	MEG	HFO detection	20 EPs	6–60	1:1	/	60 min	306	1 s	2,400 Hz	band-pass filter: 1–70 Hz, 80–500 Hz	/	/	TransHFO	92.86	100	96.15

#### 2.2.1. Spike detection

A large number of studies have shown that nearly 80% of patients with epilepsy are accompanied by abnormal neuronal discharges during the epileptic see period, which are mainly manifested in EEG waveforms as isolated spikes, spike trains, sharp waves, and spike-wave complexes (Tzallas et al., 2012; Spyrou et al., 2016). Clinically, it is common to choose to extract epileptiform spikes and spike-waves during interictal periods and to obtain epilepsy-related pathological information by quantitative analysis. Although spikes and sharp waves are clinically defined differently, in the field of automatic identification, the two are uniformly referred to as epileptic transients or spikes. The first attempt to extract epileptiform spikes from long-duration EEG signals was made by Stevens et al. (1972), which initiated the study of automatic spike detection. Since then, numerous spike detection algorithms have been generated, involving different directions such as morphology-based (Liu et al., 2012; Zhang et al., 2013), signal correlation (Lodder et al., 2013; Karacor et al., 2014; Khalid et al., 2017), sub-band decomposition (Casson and Rodriguez-Villegas, 2009) and feature engineering correlation (Oikonomou et al., 2007; Azami and Sanei, 2014; Young et al., 2018). Although there is no formal definition of MEG spikes to date (Nowak et al., 2009) and direct application of the definition of spikes in EEG may not always be applicable (Kakisaka et al., 2013), spikes and spikes in the MEG signal can be used to assist in the diagnosis of epilepsy. Compared to EEG spikes, MEG spikes are typically shorter in duration and have a steeper rise frequency (Ossenblok et al., 2007), and the MEG signal also has a higher SNR than EEG for more superficial sources, suggesting that MEG is more suitable for accurate localization of neocortical epileptic sources (Hillebrand and Barnes, 2002; Goldenholz et al., 2009). As a result, interictal MEG is increasingly being used for the preoperative assessment of epilepsy. MEG localization of interictal spike-wave regions has been shown to be in good agreement with intracranial video EEG (Knowlton and Shih, 2004; Knowlton, 2008). The most common clinical spike-wave detection is manual determination of the MEG signal by an experienced neurophysiologist. However, the subjective review process is very time-consuming (Wilson and Emerson, 2002) and the results can vary between experts (Scheuer et al., 2017).

We briefly summarize the studies on spike detection using CAD methods. Ossadtchi et al. was the first to detect spike components from multichannel MEG signals using independent component analysis (ICA) and clustering. This study laid the foundation for the subsequent research on MEG-based spike detection (Ossadtchi et al., 2004). Khalid et al. used common spatial patterns (CSP) to extract spike features and used LDA for classification and achieved average sensitivity and specificity of 91.03% and 94.21% (Khalid et al., 2016b). Alotaiby et al. used statistical features and genetic programming (GP) with the K-nearest neighbor (KNN) for interictal spike detection in MEG signal, achieved an average sensitivity and specificity of 91.75 and 92.99% (Alotaiby et al., 2017). Khalid et al. used dynamic time warping (DTW) to detect spikes and achieved sensitivity of 92.45% and a specificity of 95.81% (Khalid et al., 2017). Chahid et al. proposed to use the largest negative eigenfunction in the absolute value of the discrete spectrum of the Schrödinger Operator as feature, and used SVM for classification and achieved 92.51% and 89.10% average sensitivity and average specificity (Chahid et al., 2019). Chahid et al. used position weight matrix (PWM) combined with uniform quantizer method (QuPWM) for feature extraction of epileptic spike and then used RBF-SVM for classification, achieved sensitivity of 87.20%, specificity of 97.76%, and accuracy of 92.48% as the results (Chahid et al., 2020). Sdoukopoulou et al. considered a combined MEG and EEG approach to develop a multi-feature and iterative classification scheme and achieved a classification result of recall 90.2%, specificity 95.1%, and accuracy 92.8% (Sdoukopoulou et al., 2021). Kaur et al. proposed a strategy of locating spikes in the phase locking functional brain connectivity network using a machine learning method which achieved a classification accuracy of up to 93.8% (Kaur et al., 2022).

MEG deep learning-based spike detection algorithms are still in the exploration stage. Zheng et al. proposed a new multi-channel spike detection algorithm for MEG based on the deep learning framework EMS-Net (Zheng et al., 2019), achieving 91.60–99.96% accuracy, sensitivity, and specificity. Hirano et al. performed an AI-based identification for NEG interictal epileptiform discharges and estimated equivalent current dipoles equivalent current dipoles (ECD) (Hirano et al., 2022): a SE-ResNet-based classification model was designed to classify the data (Hu et al., 2018), followed by a DeepUNet-based splitting architecture (Li et al., 2018) for spike detection showed that 79.52 and 99.71% sensitivity and specificity were achieved and that the ECD was comparable to that estimated by neuroscientists.

#### 2.2.2. HFO detection

Clinical studies of HFO have made great progress in recent decades: there is a large body of research demonstrating that areas of HFO are strongly correlated with the epileptogenic zone and that HFO appears to delineate the epileptogenic focus better than spikes and can be considered as a potential marker of the epileptogenic zone. MEG uses the HFO of the interictal brain signal to localize the lesion, reducing recording time and patient distress. Because HFOs are short-lived, low-amplitude events, visual detection is very time-consuming, taking a minimum of 10 hours to mark an HFO in a 10-minute and 10-channel recording. Due to the low feasibility of manually labeling HFOs on a large scale, there is an urgent need for automated detection algorithms to assist physicians in the diagnosis and help with preoperative localization. Xiang et al. published the first study to detect HFOs in MEG of pediatric epilepsy patients in 2010 (Xiang et al., 2010). In 2016, Papadelis et al. described a method to simultaneously detect HFOs in scalp EEG and MEG signals of pediatric epilepsy patients (Papadelis et al., 2016): first, the envelope with communication signals was calculated using the Hilbert transform and the z-score of the envelope was calculated, and the z-score threshold was set to mark candidate HFOs in the time domain. Then, the Morlet transform was used to time-frequency transform the candidate HFOs, analyze the instantaneous power spectrum and check for “islands” in the time-frequency map to distinguish artifacts. Finally, the results were compared with invasive recordings. The results show that scalp EEG and MEG can detect and localize HFOs non-invasively and reliably. von Ellenrieder et al. found that (von Ellenrieder et al., 2016): pre-detection was performed when the root-mean-square value of the narrow-band amplitude was larger than the root-mean-square of the background activity. Afterward, some candidate HFOs were then discarded based on the definition that high-frequency oscillations are those in which at least four consecutive oscillations occur in a short of period time. The detection results agreed 85% with the results of the expert visual inspection. In 2017, van Klink et al. improved on the detector proposed by Burnos (Burnos et al., 2014, 2016; van Klink et al., 2017) to automatically detect Ripple in the sensor: first by calculating the Stockwell entropy to determine the baseline, and then using the Hilbert transform to calculate the envelope to determine Ripple. The automatically detected Ripple agreed with the patient’s MEG spike by 87.5%, indicating that automatic detection of MEG fluctuations in the time domain is feasible. The above study shows that the automatic detection of HFO signals in the MEG can be useful in aiding the diagnosis of patients with epilepsy. It can be seen that such algorithms are typically divided into two stages: first, extraction of features to pre-detect HFOs; and second, classification of previously detected candidate events to distinguish real HFOs from artifacts and noise in the signal. HFO feature extraction includes time-domain features, frequency-domain features, and time-frequency-domain features. Classification methods mainly include thresholding and machine learning methods. Thresholding methods are more common, but the amplitude and frequency of HFO are highly variable and a fixed threshold may limit the performance of the detector, so the thresholding method has unavoidable limitations. Machine learning methods include ordinary machine learning algorithms including SVM, LDA, and deep learning algorithms.

In the following, we will briefly summarize the research on HFO detection using deep learning methods. Guo et al. proposed a stacked sparse autoencoder-based MEG HFO detector (SMO), which achieved 88.2, 91.6 and 89.9% sensitivity, specificity and accuracy, respectively, which is the first study using deep learning approach for HFO detection of MEG signals (Guo et al., 2018). Guo et al. also proposed a method for automatic detection of ripple and fast ripple in 2020, using virtual sample generation based on adaptive synthetic, and attention neural networks to build models for classification, achieving an average accuracy of 89.3% (Guo et al., 2020). Liu et al. applied MEGNet, an improved CapsuleNet model, to classify MEG, and achieved the result of 95% accuracy, 94% recall, 94% F1-score and 94% accuracy (Liu et al., 2020). Guo et al. developed a Transformer-based model for classifying HFO (TransHFO), which combined the advantages of virtual sample generation and multi-head attention mechanism to achieve classification results with 96.15% accuracy, 100% precision, 92.86% sensitivity, and 100% specificity (Guo et al., 2022).

## 3. Discussion

We will discuss the research on epilepsy classification of MEG signal using CAD approach of Section 2.1 in Section 3.1, epilepsy-assisted localization methods using MEG signals of Section 2.2 in Section 3.2, then limitations and suggestions of the current study in Section 3.3.

### 3.1. Classification for epilepsy detection using MEG signals

The discussion in Section “2. Materials and methods” shows that the classification methods for MEG-based CAD systems are still more oriented towards machine learning, with only a few studies employing deep learning methods. This is mainly because of the complexity of MEG signals, which have relatively few features if viewed directly from the signal, especially resting-state MEGs. Moreover, deep learning requires a large number of training samples, and MEGs are not specifically or publicly available in databases due to the rarity of the data, thus limiting extensive research by academics.

In addition, some scholars have used other methods to classify epilepsy in MEG signal. Soriano et al. used the Extreme Learning Machine (ELM) machine learning method to classify patients with frontal lobe partial epilepsy, idiopathic generalized epilepsy, and healthy subjects using resting state MEG (Soriano et al., 2017): this study extracted the total and the relative power spectral densities (PSD), PLV and the phase-lag index (PLI) as features, which were then classified using ELM. The best results were obtained when using the PSD as a feature to differentiate between epileptic and healthy subjects (90% accuracy), and the best results were obtained when using the combination of PSD and PLV as input to differentiate between frontal and generalized epilepsy (93% accuracy).

Bhanot et al. labelled the brain into eight regions (left-frontal, left-occipital, left parietal, left-temporal, right-frontal, right-occipital, right-parietal and right- temporal) and locate the epileptogenic zone using a classification approach (Bhanot et al., 2022). To our knowledge, this is the first exploration of using MEG data to broadly classify epileptogenic zones. The study extracted four statistical features that are sensitive to the seizure period, that is, short-time permutation entropy (STPE), gradient of STPE (GSTPE), short-time energy (STE), and short-time mean (STM), and were classified using the RUSBoost algorithm, achieving an accuracy of 93.4%, specificity of 93%, sensitivity of 93% and area under the curve (AUC) of 0.97, respectively.

Tables 1, 2 demonstrate the methods of classification of epilepsy discussed above. As previously mentioned, the processing flow of machine learning consists of three main steps: pre-processing of the signal, finding and extracting relevant features, and inputting a classifier. The deep learning approach does not require manual extraction of relevant features, but only two steps of pre-processing and classification. While machine learning methods such as LDA, thresholding, SVM and ELM are mainly used for epilepsy classification based on MEG signals, deep learning includes the EnvNet-v2 based MNet method and the MSAM method. This is mainly because of the series of machine learning and deep learning methods that have gradually been born with the development of artificial intelligence. Deep learning will undoubtedly go for better results if there is a sufficient amount of sample data, however at this stage, machine learning can also get good classification results as long as relatively suitable features are extracted. In terms of feature selection, statistical features (Khalid et al., 2015; Alotaiby et al., 2019), phase features (Soriano et al., 2017; Matsubara et al., 2018; Gautham et al., 2022), and graph theoretical features are mainly used for the classification of MEG-based signals (Wu et al., 2018). We can see that a proportion of studies use traditional time-domain signal analysis methods, and over time, more still use frequency-domain and phase-based signal analysis methods or brain signal analysis methods, as these methods better highlight epileptic abnormalities. Alternatively, they can be divided into signal features (Khalid et al., 2015; Alotaiby et al., 2019)versus network features (Soriano et al., 2017; Matsubara et al., 2018; Wu et al., 2018; Gautham et al., 2022; Wang et al., 2022) and the results using network features are generally better than those using signal features.

### 3.2. Spike detection and HFO detection using MEG signals

Because of the advantages of MEG signals for epilepsy-assisted localization, several studies have focused in recent years on the detection of abnormal MEG epileptic signal waveforms (spike, HFO) using the CAD approach. A brief review of these studies is presented in Section “3. Discussion” and summarized in Table 3. Similarly to epilepsy detection using MEG signals, studies on spike detection, and HFO detection have mainly focused on machine learning, with only a relatively small number of studies using deep learning methods.

The spike detection algorithm based on traditional machine learning is mainly divided into three steps: (i) firstly, the MEG signal is pre-processed; (ii) secondly, features are manually extracted according to the characteristics of the spike, reducing the dimensionality of the signal while highlighting the differences between the spike and the background signal; and (iii) finally, the spike and background signal are bifurcated according to the obtained features. Deep learning, on the other hand, combines the two steps of feature extraction and classification, reducing the laborious step of manually extracting features. Similar to spike detection, HFO detection is also divided into three steps: (i) first pre-processing the signal; (ii) then pre-detecting possible HFOs; and (iii) classifying previously detected candidate events in order to distinguish real HFOs from artefacts and noise in the signal. In terms of feature selection, similar to the use of MEG signals for epilepsy detection and classification, statistical features (Alotaiby et al., 2017; Khalid et al., 2017; Sdoukopoulou et al., 2021), phase features (Kaur et al., 2022), graph theoretic/networks (Sdoukopoulou et al., 2021) and other features have been explored in the time and frequency domains. Overall, the detection of epileptogenic foci will be facilitated by the use of CAD methods for the detection of abnormal signals in epilepsy.

### 3.3. Limitations and suggestions of the current study

Most of the existing epilepsy-based classification studies are based on EEG, while only a few are based on MEG, and even fewer are based on deep learning, so we believe that the use of MEG for the classification of epilepsy is very innovative and should be exploited more to better serve clinical needs. The main problem with MEG-based psychiatric research is the small sample size and the lack of a dedicated standard database (previous studies have been based on non-public data from hospitals), which cannot be fully compensated for by sample augmentation. This is mainly due to the fact that MEG is a relatively new imaging technology with expensive operating costs and examination fees, and therefore has not yet become as popular as EEG. However, with technological advances and more favourable policies, MEG as an imaging tool will certainly become more popular in the future.

Firstly, to address the problem of insufficient sample size of MEG disease data, we are suggested to collect more data in the future, and build up a standard database and explore more data enhancement methods. Secondly, with the development of artificial intelligence and deep learning, we can conduct more MEG research based on deep learning while data are being expanded. In general, we need to do more research on MEG and thus make more use of the clinical value of MEG.

## 4. Conclusion

This paper provides a brief review and summary of the methods used to classify epilepsy on MEG data, discussing the features used in each work. It can be seen that classification based on MEG epilepsy can be summarized as using signal features as well as network features, and all have achieved good classification results. The majority of the studies used machine learning methods, with only a few using deep learning, and the results show that the accuracy of machine learning and deep learning is comparable as long as relatively appropriate features are selected. In the meantime, this review discusses the progress of research on MEG classification in epilepsy-assisted localization. Furthermore, all the methods described in this paper are supervised learning, i.e. they require labelled data for validation. Finally, we present a summary of the unresolved issues and future research directions in the field of epilepsy classification using MEG. We hope that this review will provide the reader with a general understanding of the classification problem of MEG and provide ideas and directions for future research.

Despite the tremendous efforts of these studies to aid in the diagnosis and localization of epilepsy, the algorithms developed to date are still not as reliable as those developed by experienced neuroscientists. Therefore, the development of a useful and reliable automated diagnostic system will require the efforts of the scientific community, and in addition, the creation of a standard MEG epilepsy database would be a huge advancement for this type of research.

## Author contributions

RP and CY conducted the literature search and prepared the initial draft of the manuscript, edited the final draft of the manuscript. ZL, JR, and YD involved in study conception and contributed to intellectual content. All authors contributed to the article and approved the submitted version.
